# Evaluation of Partial Volume Correction Techniques for Sodium MRI of the Achilles Tendon

**DOI:** 10.1002/mrm.70208

**Published:** 2025-11-27

**Authors:** Rika Möller, Benedikt Kamp, Paula Leja, Thomas A. Thiel, Eric Bechler, Hans‐Jörg Wittsack, Gerald Antoch, Armin M. Nagel, Lena M. Wilms, Miriam Frenken, Anja Müller‐Lutz

**Affiliations:** ^1^ Medical Faculty, Department of Diagnostic and Interventional Radiology University Dusseldorf Dusseldorf Germany; ^2^ Core Facility for Magnetic Resonance Imaging, Medical Faculty and University Hospital Düsseldorf Heinrich‐Heine‐University Düsseldorf Düsseldorf Germany; ^3^ Institute of Radiology, University Hospital Erlangen Friedrich‐Alexander‐Universität Erlangen Nürnberg (FAU) Erlangen Germany; ^4^ Divison of Medical Physics in Radiology German Cancer Research Center (DKFZ) Heidelberg Germany

**Keywords:** ^23^Na‐MRI, Achilles tendon, partial volume correction, sodium concentration, sodium MRI

## Abstract

**Purpose:**

To evaluate partial volume correction (PVC) techniques for sodium MRI of the Achilles tendon in situ and in vivo.

**Methods:**

Five PVC methods were evaluated including a volume ratio of the proton and sodium segmentations (PSSR), a modified least trimmed square (3D‐mLTS) linear regression, a geometric transfer matrix (GTM) approach, a single target correction (STC), and a novel estimated single target correction (eSTC). Their performance was tested using simulated data and 3 T MR data of two volunteers' Achilles tendons acquired at different resolutions: 1.5, 2.0, 3.0, and 4.5 mm^3^. Since there was no in vivo ground truth, the highest‐resolution apparent tissue sodium contents (aTSC) were used.

**Results:**

In the simulation, all PVC methods reduced the difference between the actual and calculated concentrations and were 11.69 ± 6.17 mM without PVC, 4.90 ± 5.40 mM with the PSSR, 4.86 ± 5.19 mM with the mLTS, 1.72 ± 4.13 mM with the GTM, 0.36 ± 1.77 mM with STC and 0.26 ± 1.63 mM with the eSTC. In vivo, the difference in aTSCs between the lower and the highest resolution decreased with all PVCs ranging from 3.6 to 38.8 mM without PVC, 2.8–20.4 mM with PSSR, 4.5–25.9 mM with mLTS, 0.9–7.8 mM with GTM, 0.1–23.8 mM with STC, and 0.7–7.7 mM with eSTC.

**Conclusion:**

PVC generally improved the accuracy of aTSC calculations. The newly introduced eSTC produced the most accurate results for the Achilles tendon.

## 1 Introduction

Sodium MRI is a functional imaging technique that provides physiological information about the human body [1]. In the musculoskeletal system, it is often used to evaluate tissue viability in areas such as cartilage [2], intervertebral discs [3, 4], and tendons [5, 6], because it correlates with glycosaminoglycan content [2, 7], which is an early indicator of pathologies [8, 9]. Achilles tendinitis is one of the most common sports injuries [10]. Since it is challenging to treat [11] and many patients experience long‐lasting functional deficits [12], early detection is particularly important. Previous studies have demonstrated the ability of sodium MRI to assess Achilles tendinopathies, both ex vivo [5] and in vivo [6, 13, 14, 15], using sodium SNR [14] and apparent tissue sodium content (aTSC) [6].

There are limitations of sodium MRI. Due to the lower inherent sodium concentration in the human body [16] and lower NMR sensitivity [17] compared to ^1^H, large voxel sizes are necessary to obtain an acceptable SNR. Furthermore, the ^23^Na‐nucleus is characterized by fast biexponential transversal relaxation [18]. Thus, radial sequences with ultrashort echo times [19] are widely used [20]. Large voxel sizes and radial acquisitions lead to two different forms of partial volume effects (PVE) referred to as the tissue‐fraction effect and the spill‐over effect [21]. The tissue‐fraction effect arises from low resolutions when several tissues are present in the same voxel, leading to intravoxel signal averaging. The spill‐over effect originates from the point spread function (PSF) of imaging systems, leading to signal spreading into adjacent voxels [21]. Due to its geometry, radial k‐space sampling results in PSFs with larger FWHM values compared to Cartesian sampling [22]. Various partial volume correction (PVC) techniques exist for both effects and have been applied to sodium brain data [23, 24, 25], heart [26], skin [27], and cartilage [28]. However, these methods have not yet been compared to each other for any application or the Achilles tendon (AT) in particular [6].

Therefore, this study aimed to evaluate and compare five correction techniques to determine the performance of PVC for their application to the AT with a density adapted 3D radial (DA‐3D‐RAD) [19] sequence: a region correction of tissue‐fraction artifacts based on the proton‐to‐sodium segmentation ratio (PSSR) [28], a modified least trimmed square (3D‐mLTS) linear regression [25], a geometric transfer matrix (GTM) [23], a single target correction (STC) [29], and a novel modified estimated single target correction (eSTC). The first two methods correct tissue‐fraction artifacts, while the rest correct spill‐over. All methods were tested using simulated images with Monte Carlo simulations and an in vivo evaluation for application to the AT. The study hypothesis was that PVC could improve aTSC determination.

## 2 Methods

Five PVC techniques were evaluated using a simulation and in vivo imaging. In the simulation, the methods were tested for their ability to restore the ground truth data from a PVE‐corrupted dataset. Since there was no in vivo ground truth available, four images of the same volunteer were acquired at different spatial resolutions. The premise was that the PVEs would be more severe in lower resolution images, so the highest‐resolution results were treated as the ground truth.

### 2.1 Proton‐To‐Sodium Segmentation Ratio (PSSR)

Most segmentation masks for sodium imaging are created using higher‐resolution proton images that are then scaled down to fit the sodium image. This results in tissue‐fraction effects at the edges, because the voxels of the sodium image are larger and include signals from outside of the original segmentation. Müller‐Lutz et al. [28] used the volume ratio *v*_*PS*_ between the two versions of the segmentation as a weighting factor to remove the influence of additional signals. This was developed for wrist cartilage [28], where the surrounding bones are assumed to have almost zero sodium signal. However, since the surroundings of the AT include sodium‐containing tissues like skin or muscle [27, 30, 31], the formula was adapted. When *S*_*m*_ is the measured mean signal intensity in the region of interest (ROI) and *S*_*sur*_ the mean signal of the surroundings, the corrected mean signal *C* can be calculated as follows:

(1) $$C=\left[\frac{S_{m}-\left(S_{\mathrm{sur}}\cdot \left(1-v_{\mathrm{PS}}\right)\;\right)}{v_{\mathrm{PS}}}\right]$$

The factor (1—*v*_*PS*_) represents the volume fraction outside of the high‐resolution segmentation. The signal of the immediate surroundings *S*_sur_ is estimated by calculating the mean sodium signal of the voxels immediately surrounding the ROI.

### 2.2 Modified Least Trimmed Square (3D‐mLTS) Linear Regression

Asllani et al. [32] first introduced the concept of correcting tissue‐fraction effects on a voxel‐by‐voxel basis for arterial spin labeling MRI. Using a high‐resolution segmentation image, one can calculate the fraction of each tissue type within a voxel of a lower‐resolution image. Assuming constant signal intensities for each tissue type within a kernel centered at a voxel, the original signal intensities of that voxel can be recovered using linear regression [32]. To minimize signal smoothing, Liang et al. [33] extended the method by defining a subset of voxels within the kernel to calculate the intensities. Residuals are calculated from this subset, which is repeatedly redefined until convergence is reached [33]. Kim et al. [25] modified the method for application to sodium MRI by using 3D kernels of 3 × 3 × 3 voxels and adding a trimming parameter of 0.4 to define the size of the subsets [25].

### 2.3 Geometric Transfer Matrix (GTM)

The geometric transfer matrix (GTM) approach was originally developed for PET imaging and can correct spill‐over artifacts region‐wise [34]. Niesporek et al. [23] adapted this method for sodium MRI by simulating the PSF using the known k‐space trajectory of the DA‐3D‐RAD sequence and the T2* decay [19, 35]. After obtaining the PSF, the region spread function (RSF) of the individual tissue compartments can be calculated by convolving the PSF with the segmentation. Then, the spill‐over contributions of each compartment are accumulated into weighting factors. These factors are arranged into a set of linear equations that form the GTM. Subsequently, the corrected mean tissue intensities can be recovered by matrix inversion [23].

### 2.4 Single Target Correction (STC)

Erlandsson et al. [29, 36, 37, 38] have corrected spill‐over artifacts iteratively on a voxel‐by‐voxel basis for PET applications. Their method corrects the PVEs between a single target and its surroundings. In each iteration, the image is corrected by subtracting the spilled‐over signal from the surroundings into the target tissue, and vice versa. These spilling contributions are calculated by convolving the signal of either compartment with its PSF and isolating the contribution to the other compartment. Then, the image is divided by a recovery factor R, which corrects for the spilled‐out signal [36] (equivalent to the sum of the individual RSFs, inside their corresponding tissue mask **m**_*i*_) $R=\sum_{i}{\mathrm{RSF}}_{i}$, where $\mathrm{RS}F_{i}=m_{i}\cdot \left(m_{i}*\mathrm{PSF}\right)$. The resulting image is an estimate to recalculate the spilled‐over signals and the process is repeated until convergence.

The algorithm is described in detail including a pseudocode by Sari et al. [36] and was adapted for sodium MRI by adding the simulated PSFs from Niesporek et al. [23] To fulfill the non‐negativity condition, all negative values are replaced by their nearest minimal positive neighbor in each iteration.

### 2.5 Estimated Single Target Correction (eSTC)

**FIGURE 1:**
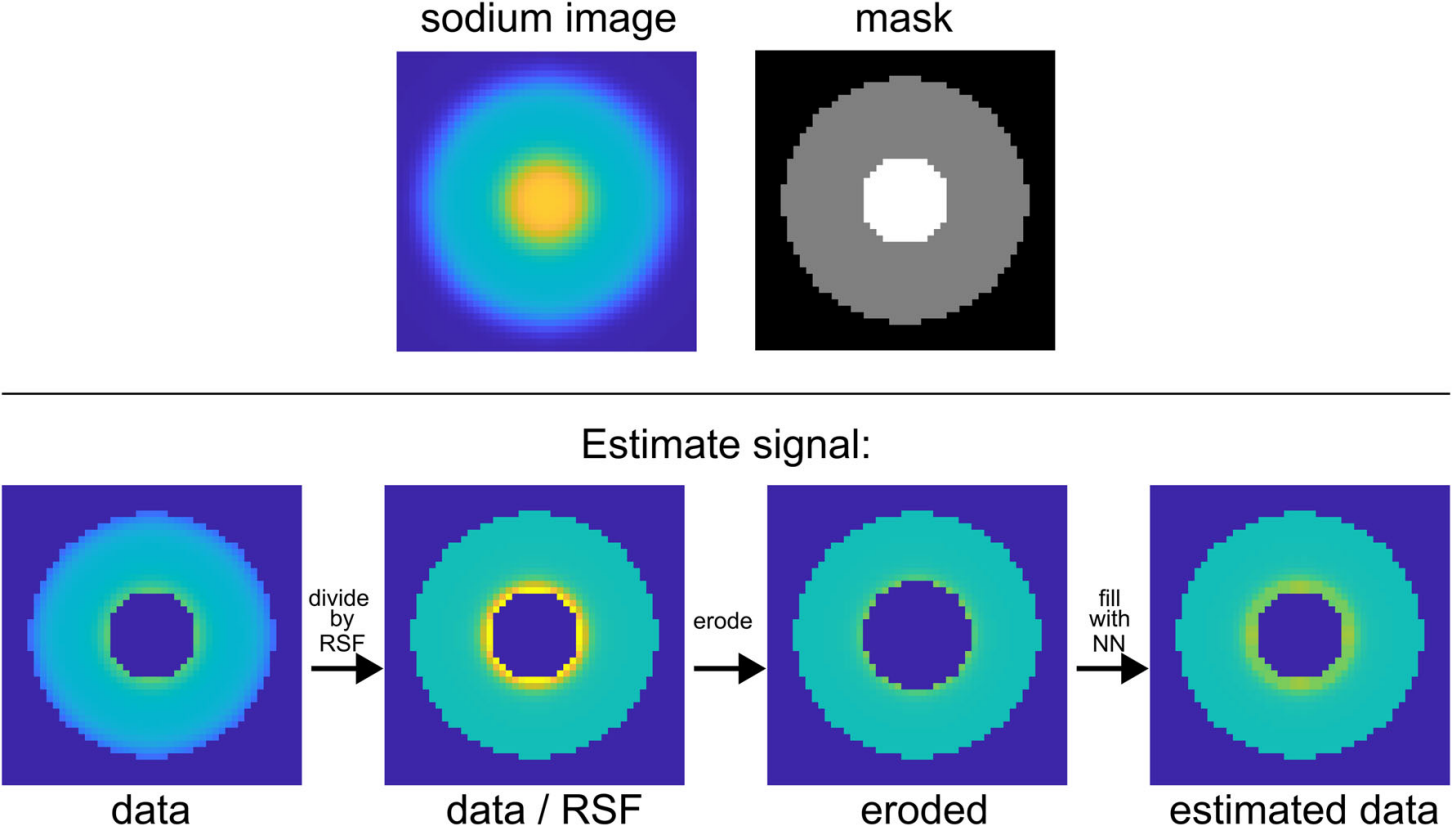
Principle of estimating sodium distributions with the example of a two‐compartment image. As illustrated above, a PVE corrupted sodium image is presented, encompassing both the inner and outer parts, along with their respective masks. The row below presents the estimation of the actual signal distribution with the surrounding data. Initially, the data is isolated by applying its mask and the spill‐out is corrected by dividing it by its RSF. The overestimated edge voxels are eroded and then filled back up with their nearest neighbor (NN).

The STC method [39] relies on repeated convolution to calculate spill‐over contributions, resulting in lengthy computation times. To address this issue, this study proposes an alternative method for estimating the contributions. It assumes that PVEs predominantly occur at tissue borders. Similar to the STC, the object is separated into targets *i* and their surroundings *sur*:

1. First, the surrounding signal is divided by its RSF, leading to overestimation of edge voxels. To eliminate this, the edges of the surroundings are eroded, and the missing voxels are replaced by their nearest neighbor (Figure 1). The estimated surrounding signal ***E***_sur_ arises from
(2) $$E_{\mathrm{sur}}=r\left(\frac{m_{\mathrm{sur}}\cdot S}{\mathrm{RS}F_{\mathrm{sur}}}\right),$$
  where *r* represents the erosion of the data and its replacement with the nearest neighbors, while ***S*** is the image signal, ***m***_sur_ the mask of the surroundings and **RSF**_sur_ the corresponding RSF.
2. To estimate the tissue signals ***E***_*i*_, the spill‐in from the surroundings is removed prior to dividing by the RSF. Spill‐in of ***E***_sur_ is calculated by convolving it with its PSF and defining its contribution to compartment *i* by applying the mask of that compartment ***m***_*i*_
(3) $$E_{i}=r\left(\frac{S_{i}-\left[m_{i}\cdot \left(E_{\mathrm{sur}}*\mathrm{PS}F_{\mathrm{sur}}\right)\right]}{\mathrm{RS}F_{i}}\right)$$
**S**_*i*_ is the measured signal of the compartment and **RSF**_*i*_ is the RSF inside the mask.
3. These estimated values ***E***_*i*_ are used to calculate all spill‐over contributions. Next, the corrected signal distribution of one tissue ***C***_*i*_ is calculated by subtracting all spilled‐in signals from its measured signal and dividing by its RSF
(4) $$C_{i}=\frac{1}{\mathrm{RS}F_{i}}\left[S_{i}-\sum_{\begin{array}{l} i\\ j\neq i \end{array}}m_{i}\cdot \left(E_{j}*\mathrm{PS}F_{j}\right)\right]$$
  After calculating a corrected compartment, its estimated value ***E***_*i*_ is updated by ***E***_*i*_ = *r*(***C***_*i*_) for the calculation of the next compartment. This is repeated for all compartments, starting with the largest and ending with the smallest. Negative values are replaced by their nearest neighbor. This approach eliminates the need for more than one iteration.

### 2.6 Simulation

**FIGURE 2:**
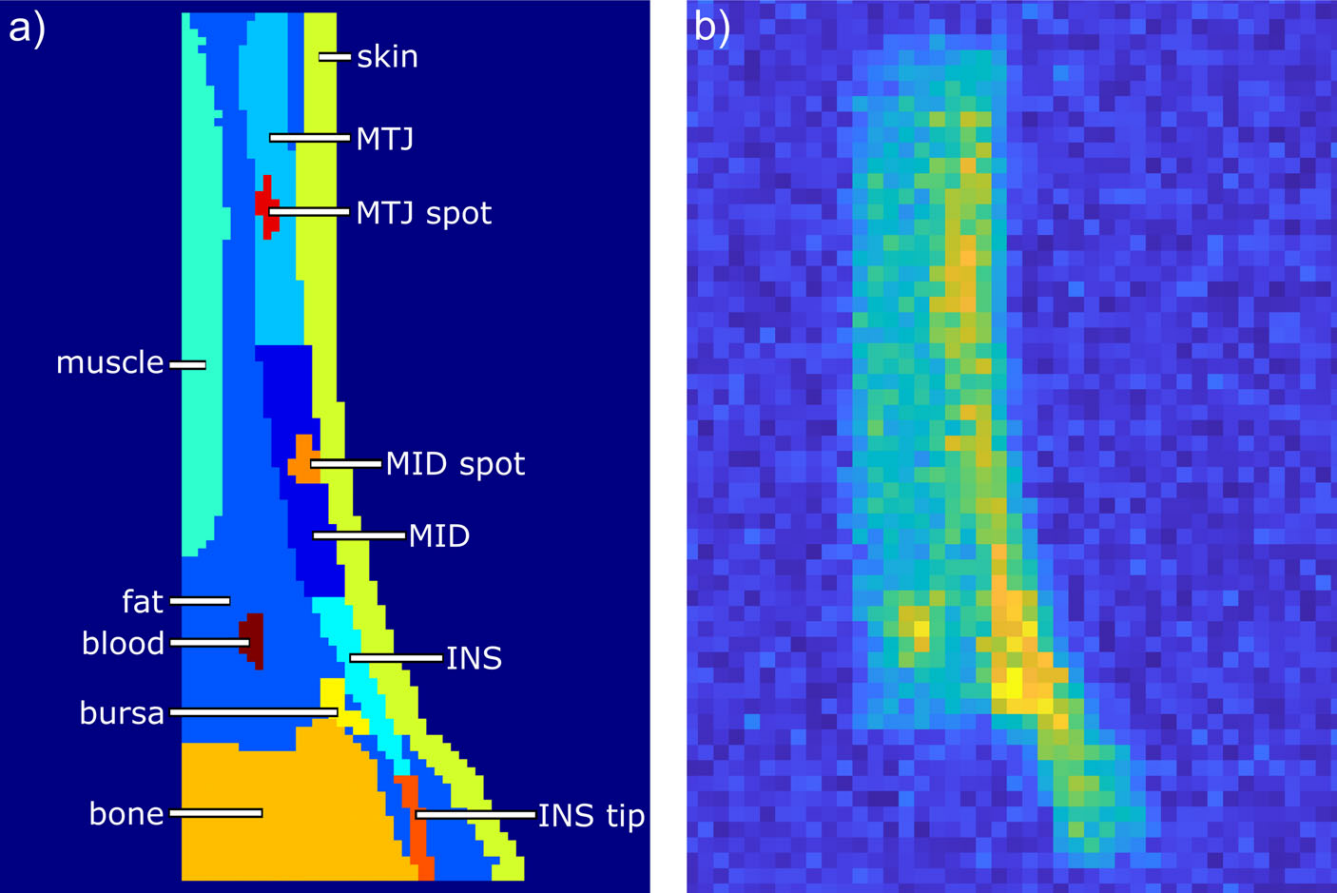
(a) Segmentation of the Achilles tendon and surrounding tissues including spots to emulate signal inhomogeneities. The corresponding tissues are color coded and indicated in the legend. (b) simulated sodium image including concentrations, relaxation times, downsizing, coil sensitivity, PSFs and noise at an SNR of 10 in the MID.

A 3D artificial dataset was produced by segmenting a proton image of an Achilles tendon and its surrounding tissues with a resolution of 1 × 1 × 1 mm^3^. These tissues included the AT, the skin, fat, the soleus and gastrocnemius muscles, the retrocalcaneal bursa, some blood vessels and the calcaneus. The AT was divided into the insertion point into the calcaneus (INS), the middle portion of the tendon (MID), and the myotendinous junction (MTJ). A concentration was assigned to each tissue except for the calcaneus, as bone has a negligible sodium content. Additionally, two spots with higher and lower concentrations were added to the AT to model regional inhomogeneities. The tip of the INS was given a higher concentration to model the observed signal increase towards the tip [6, 14]. The concentrations in the Achilles tendon were chosen to reflect the magnitude of in vivo measurements. The segmented image is shown in Figure 2a. Four reference phantoms with a 4% agarose concentration were included for calibration. This concentration image was used as the ground truth.

In the absence of optimal acquisition parameters, such as ultrashort echo times and long repetition times, measuring sodium signals is susceptible to a phenomenon known as relaxation weighting. To simulate the subsequent reduction in signal, each tissue was multiplied by an exponential weighting factor, resulting from the tissues T_1_, T_2l_*, and T_2s_* with a TR of 15 ms and a TE of 0.1 ms [6]. These values were chosen to resemble the in vivo acquisitions (Section 2.7). All concentrations and relaxation times are listed in Table 1. The image was then downscaled to a (2 × 2 × 2) mm^3^ resolution to simulate the tissue‐fraction effect by calculating the mean value of the corresponding smaller voxels. After downsizing, the sensitivity of the sodium coil (Section 2.7) was applied by multiplying the normalized image of a homogeneous phantom [4, 6, 28].

**TABLE 1:** Sodium concentrations and relaxation times of all simulated tissues, including their sources.

Tissue	Concentration [mM]	T_1_ [ms]	T_2l_* [ms]	T_2s_* [ms]	Source concentration	Source relaxation times
INS	25	18.4	14.5	1.4	—	2022 Kamp [6]
MID	15	19.2	14.2	1.4	—	2022 Kamp [6]
MTJ	18	23.3	14.6	1.5	—	2022 Kamp [6]
INS tip	30	18.4	14.5	1.4	—	2022 Kamp [6]
MID spot	18	19.2	14.2	1.4	—	2022 Kamp [6]
MTJ spot	14	23.3	14.6	1.5	—	2022 Kamp [6]
Skin	34.2	27	7.6	0.5	2024 Zhu [40]	2015 Linz [27]
Fat	13.1	25.2	14.3	1.4	2020 Crescenzi [31]	2012 Madelin [41]
Muscle	20.3	25.2	14.3	1.4	2020 Crescenzi [31]	2012 Madelin [41]
Synovial fluid	140	62	28	—	2016 Zbýň [42]	2016 Zbýň [42]
Blood	81	38.4	15.8	2.0	2019 Lott [26]	2012 Madelin [41]
Agarose phantoms	50/75/100/125	38.5	13.0	6.0	2022 Kamp [6]	2022 Kamp [6]

*Note*: The concentrations in the Achilles tendon were chosen to reflect the magnitude of the in vivo measurements below, as well as previous pilot measurements of aTSCs of the Achilles tendon. The increased signal towards the INS has been observed previously [14].

Abbreviations: aTSC, apparent tissue sodium content; INS, insertion point into the calcaneus; MID, middle portion of the tendon; MTJ, myotendinous junction.

To generate signal spill‐over, PSFs were simulated using Niesporek et al.'s simulation [23] for all tissues and their relaxation times. For voxels containing more than one tissue, and therefore more than one set of relaxation times, the mean T_1_, T_2l_*, and T_2s_* relaxation times for each combination were used. The PSFs were then convolved with their corresponding voxels. Rician noise [43] was added to reflect a realistic and low SNR of the Achilles tendon of about 10 and 5 in the MID [6]. The resulting image with an SNR of 10 is shown in Figure 2b. For applications of the GTM, STC, and eSTC methods, the resulting image was zerofilled to the ground truth resolution. Concentrations were calculated for each PVC method using the post‐processing pipeline described below (Section 2.8), once without noise, then with Monte Carlo simulations with 100 iterations for both noise levels. The ground truth image was subtracted voxel‐wise from the resulting concentration maps and the mean difference and SD were calculated. Additionally, the root mean square error (RMSE) was calculated over all voxels in the AT.

### 2.7 Image Acquisition and Reconstruction

All images were acquired on a Siemens 3 T MRI (Siemens MAGNETOM Prisma, Siemens Healthineers, Erlangen, Germany) with a double‐tuned ^1^H/^23^Na surface coil (RAPID Biomedical GmbH, Rimpar, Germany) and Nagel et al.'s [19] density‐adapted 3D radial (DA‐3D‐RAD) sequence [19] with a FOV of 180 × 180 × 180 mm^3^. The ^1^H images used for segmentation were acquired with an isotropic resolution of 0.5 mm^3^ at a TR/TE of 10/6 ms, with 50 000 projections and a 10° flip angle, resulting in an acquisition time of 8:20 min. Four sodium images of two healthy volunteers (male, age: 27 a; female age: 24 a) were acquired with isotropic resolutions of 1.5, 2.0, 3.0, and 4.5 mm^3^. The same TR/TE of 15/0.1 ms and 90° flip angle were used for all sodium images, along with a pulse duration of 0.16 and a 5 ms readout time. The remaining imaging parameters are listed in Table 2. The number of projections and averages were varied to attempt SNR alignment. The volunteers were imaged supine, head first with the surface coil placed under the center of the AT. In line with previous studies, four reference phantoms (1 cm diameter, 3.5 cm height) with ^23^Na concentrations of 50, 75, 100, and 125 mM and 4% agarose content by weight (ROTI Garose, Carl Roth GmbH & Co. KG, Karlsruhe, Germany) were placed behind the coil for aTSC calculations [2, 6, 28]. Written consent was obtained from the volunteers and Ethics approval was received from the Ethics Committee, Medical Faculty of the Heinrich‐Heine‐University Düsseldorf (study number 2021‐1393_1).

**TABLE 2:** Acquisition parameters of the sodium images with the density‐adapted 3D radial (DA‐3D‐RAD) sequence and the ^23^Na/^1^H surface coil.

	^23^Na image 1.5	^23^Na image 2.0	^23^Na image 3.0	^23^Na image 4.5
Resolution [mm^3^]	1.5 × 1.5 × 1.5	2 × 2 × 2	3 × 3 × 3	4.5 × 4.5 × 4.5
Number of projections	40 000	50 000	33 330	22 220
Averages	3	1	1	1
Matrix size	120 × 120 × 120	90 × 90 × 90	60 × 60 × 60	40 × 40 × 40
Acquisition time [min:s]	30:00	12:30	8:20	5:40

Since the ^1^H/^23^Na coil is a surface coil, the measurable signal strength decreases with increasing distance. To generate weighting factors needed to correct for this decrease, an image of a homogeneous water phantom with a 154 mM ^23^Na concentration was acquired. The imaging parameters of the sensitivity profile can be found in the study from Kamp et al. [6].

All images were reconstructed offline using a custom script (MATLAB, The MathWorks Inc., Natick, USA, R2022a) [19] with a FOV of 180 × 180 × 180 mm^3^ and a Hanning filter to reduce Gibbs ringing. For further post‐processing, the sodium images were reconstructed twice, once without zerofilling to their respective matrix sizes and once zerofilled to the 0.5 mm^3^ resolution of the proton image. If necessary, the images were motion corrected by manual registration using the software ITK‐snap (version 3.8.0, Cognitica, Philadelphia, PA, USA) [44].

### 2.8 Image Post‐Processing

The ^1^H‐image was manually segmented using the software ITK‐snap (version 3.8.0, Cognitica, Philadelphia, PA, USA) [44]. The Achilles tendon was divided into the INS, MID and MTJ with a length of 3 cm each along the transversal axis [5, 6, 14]. All surrounding tissues within the coil sensitivity were segmented, including the skin, the fat, the soleus and gastrocnemius muscles, the retrocalcaneal bursa, and cartilage for the GTM application. Additionally, ROIs were defined for each of the four reference phantoms. The relaxation times used for the PSF generation were the same as those used in the simulation and are listed in Table 1. To generate the cartilage PSF, the T_1_, T_2l_*, and T_2s_* values were 14.5, 12.6, and 0.4 ms, respectively [2].

Calculation of aTSCs was conducted using a custom script (MATLAB, The MathWorks Inc., Natick, USA) for each of the five PVCs and without PVC. For evaluations without PVC, with the PSSR and the mLTS, the images without zerofilling were used, since the correction techniques are based on the size difference between the segmentation and sodium image [25, 28]. In these cases, the ROI was downsized to match the sodium image resolution. For the GTM, STC, and eSTC the zerofilled images were used.

The remaining postprocessing steps were the same for all methods. First, the PVC was applied, followed by sensitivity correction for the tissues and reference phantoms. The order of the corrections was chosen on the premise that PVEs consist of the measurable signal, which is limited by the sensitivity of the used surface coil. The sensitivity profile was manually registered to the image by matching markers that define the coil position in both the sensitivity profile and in vivo images. If necessary, the profile was resized to the corresponding resolution. Next, the reference phantoms were linearly fitted to their known concentrations and the fit results were used to calculate the aTSCs [45]. Additionally, the tissue and reference phantom signals were corrected for different relaxation times [45] using the values found in Table 1. The aTSCs of each part of the tendon were calculated, as well as a combined value by using the average signal and average relaxation times of the entire tendon. aTSC maps were calculated for all PVC methods. To generate aTSC maps for the region‐wise methods, such as the PSSR and GTM, the ratio of the corrected to uncorrected mean values was applied as a weighting factor to all voxels in the corresponding ROI.

## 3 Results

### 3.1 Results Simulation

**FIGURE 3:**
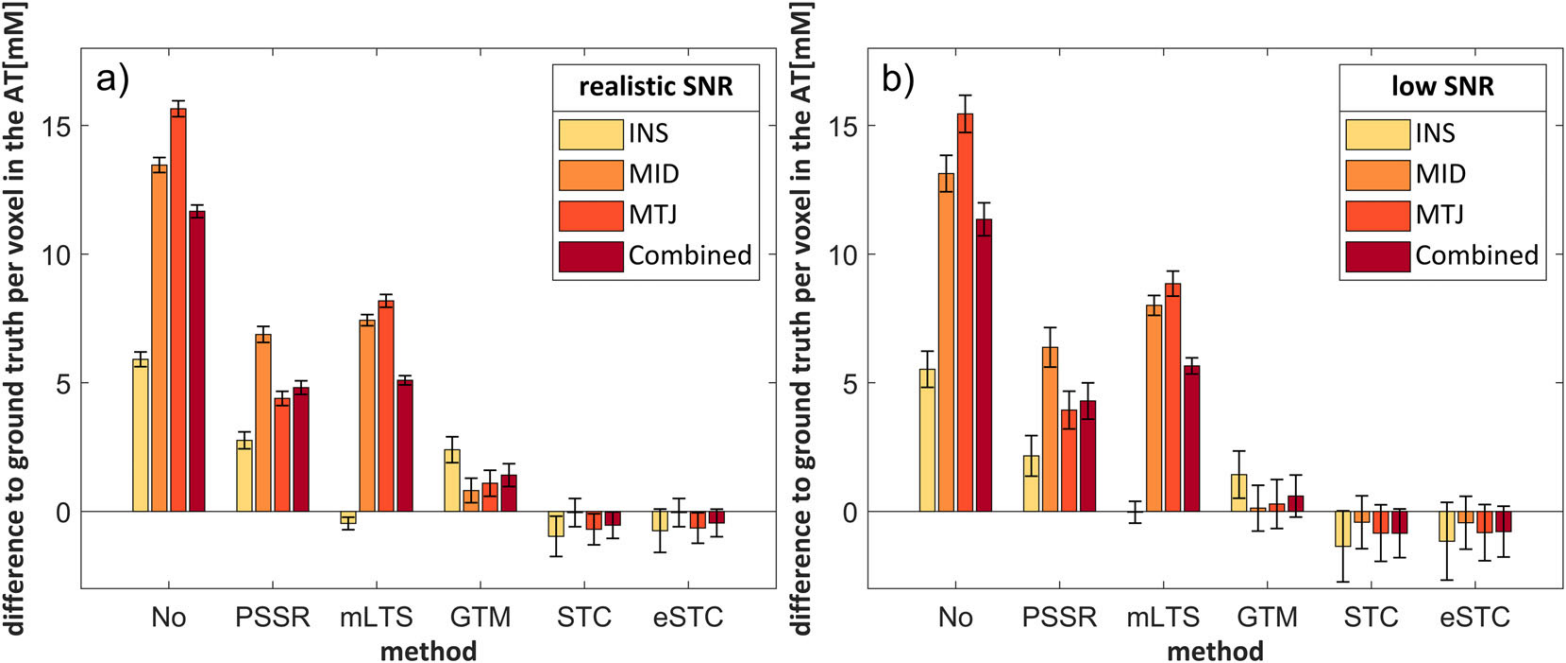
Mean differences per voxel in the Achilles tendon (AT) between the partial volume corrected image and the ground truth at an SNR in the MID of 10 (a) and 5 (b). The ground truth was voxel‐wise subtracted from the image before averaging over the respective ROI. The standard deviations over the 100 noise iterations are indicated.

Each correction method was applied individually to the simulated image, once without noise and in each of the Monte Carlo iterations. Figure 3 and Table 3 show the mean differences per voxel to the ground truth image, averaged over the Monte Carlo simulations for an SNR of 10 (a) and 5 (b). The ground truth was subtracted from the corrected image, thereby assigning positive values to instances of overestimation and negative values to instances of underestimation.

**TABLE 3:** Mean differences per voxel in the Achilles tendon (AT) between the partial volume corrected image and the ground truth at an SNR in the MID of 10 (a) and 5 (b).

PVC	Δ INS [mM]	Δ MID [mM]	Δ MTJ [mM]	Δ Combined [mM]
*SNR 10*				
No	5.91 ± 0.29	13.46 ± 0.29	15.65 ± 0.31	11.66 ± 0.25
PSSR	2.76 ± 0.33	6.88 ± 0.31	4.39 ± 0.28	4.81 ± 0.26
mLTS	−0.47 ± 0.24	7.43 ± 0.22	8.18 ± 0.25	5.10 ± 0.18
GTM	2.40 ± 0.50	0.81 ± 0.47	1.09 ± 0.51	1.41 ± 0.44
STC	−0.97 ± 0.78	−0.04 ± 0.55	−0.69 ± 0.60	−0.53 ± 0.51
eSTC	−0.75 ± 0.84	−0.04 ± 0.55	−0.64 ± 0.59	−0.45 ± 0.54
*SNR 5*				
No	5.52 ± 0.70	13.13 ± 0.71	15.45 ± 0.72	11.35 ± 0.64
PSSR	2.16 ± 0.79	6.38 ± 0.77	3.94 ± 0.74	4.29 ± 0.71
mLTS	0.03 ± 0.42	8.01 ± 0.39	8.85 ± 0.49	5.66 ± 0.32
GTM	1.43 ± 0.92	0.13 ± 0.89	0.29 ± 0.95	0.60 ± 0.82
STC	−1.36 ± 1.38	−0.42 ± 1.03	−0.84 ± 1.10	−0.85 ± 0.95
eSTC	−1.16 ± 1.51	−0.44 ± 1.03	−0.82 ± 1.09	−0.78 ± 0.99

*Note*: The ground truth was voxel‐wise subtracted from the image before averaging over the respective ROI. The standard deviations over the 100 noise iterations are given.

Abbreviations: eSTC, estimated single target correction; GTM, geometric transfer matrix; mLTS, modified least trimmed squares; PSSR, proton‐to‐sodium segmentation ratio; PVC, partial volume correction; STC, single target correction.

All correction methods reduced the differences to the ground truth. Most concentrations were overestimated, especially without PVC. Methods that corrected for tissue‐fraction effects produced larger differences than the methods that corrected for spill‐over. The STC reached convergence at SNR 10 after 13–31 iterations and at SNR 5 after 14–31. The mean differences were similar, but the SDs increased with lower SNR, especially with the spill‐over methods. The eSTC produced the smallest differences at a realistic SNR, though it was similar to the STC at both SNRs. At a low SNR all spill‐over corrections produced similar results. The overall RMSE of the Monte Carlo simulations was about 14 mM (14 mM) at SNR 10 (SNR 5) without PVC and reduced to 8 mM (9 mM) with the PSSR and mLTS. The GTM produced an RMSE of 5 mM (6 mM), while the STC and eSTC both produced an RMSE of 5 mM (9 mM). Without noise, the RMSE was the same as with an SNR of 10 for all methods except for STC and eSTC, which had an RMSE of 2 mM.

**FIGURE 4:**
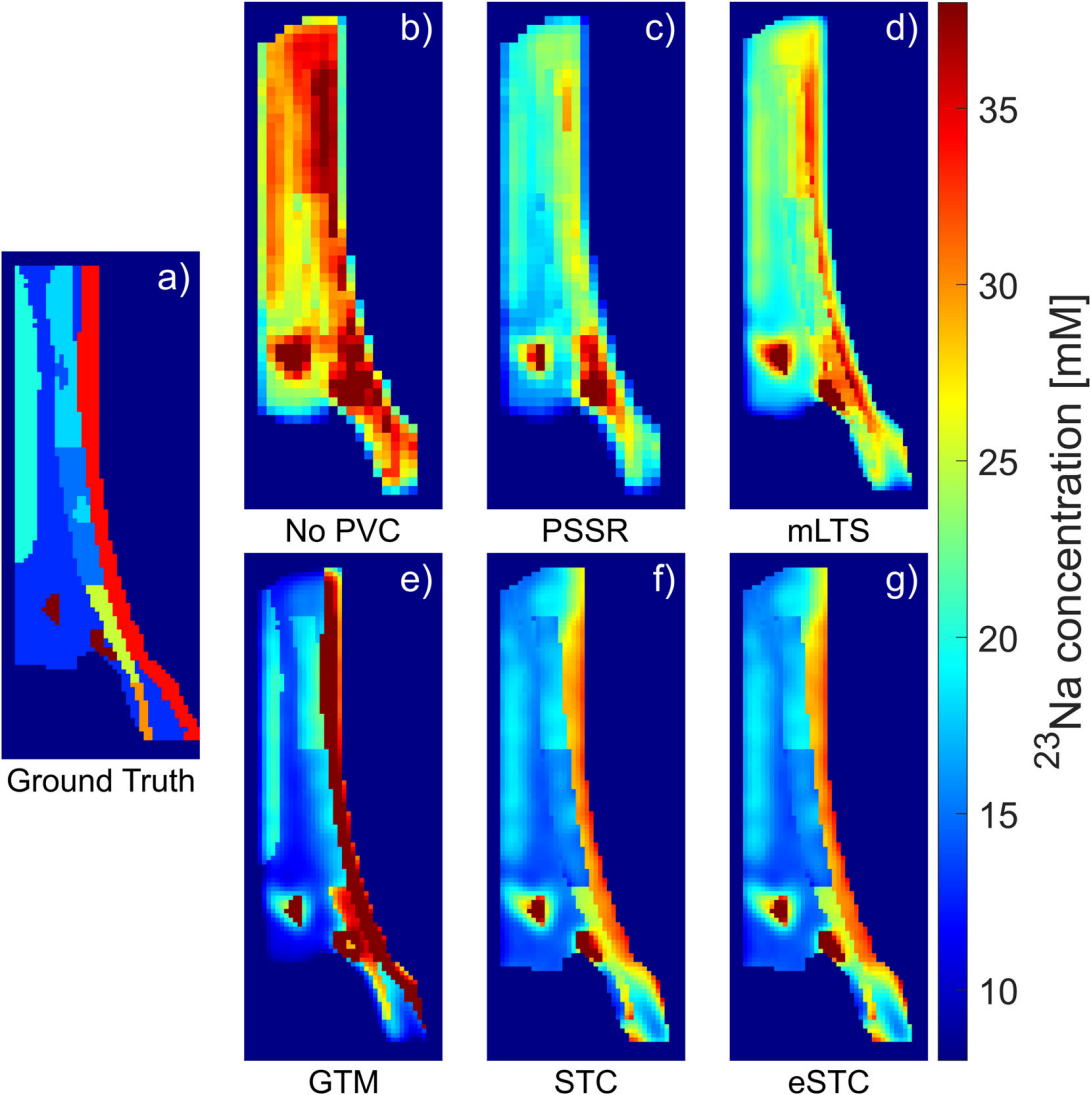
Simulated sodium concentration maps. Shown are the sagittal views of the ground truth concentration distribution (a), the calculated concentrations without PVC (b), with the PSSR (c), the mLTS (d), the GTM (e), the STC (f) and the eSTC (g). The region‐wise methods PSSR and GTM have been applied by multiplying the correction factor for each region onto each voxel in that region.

Table 4 lists the same voxel‐wise differences, but for noise‐free data. The SDs represent the variation of differences per voxel over the tendon subsections. Given that the results were obtained by means of voxel‐wise subtraction, the SDs absent of noise reflect the ability to preserve the regional differences within the AT (Table 1). These SDs were similar without PVC and the tissue‐fraction corrections, but smaller with the spill‐over corrections. Notably, the STC and eSTC had lower SDs. Figure 4 shows aTSC maps for visual representation.

**TABLE 4:** Mean differences per voxel (Δ) between the PVE corrected image without noise and the ground truth.

PVC	Δ INS [mM]	Δ MID [mM]	Δ MTJ [mM]	Δ Combined [mM]
No	5.96 ± 7.23	13.49 ± 2.50	15.64 ± 2.78	11.69 ± 6.17
PSSR	2.90 ± 8.44	6.95 ± 2.45	4.45 ± 1.69	4.90 ± 5.40
mLTS	−0.59 ± 5.56	7.17 ± 1.78	7.84 ± 2.24	4.86 ± 5.19
GTM	2.77 ± 6.70	1.08 ± 1.56	1.41 ± 1.88	1.72 ± 4.13
STC	0.76 ± 2.31	0.13 ± 0.98	0.55 ± 1.74	0.36 ± 1.77
eSTC	0.57 ± 2.09	−0.15 ± 0.96	0.45 ± 1.64	0.26 ± 1.63

*Note*: Positive values represent overestimated concentrations, negative values underestimated concentrations. The standard deviation over the respective ROI is given.

Abbreviations: eSTC, estimated single target correction; GTM, geometric transfer matrix; mLTS, modified least trimmed squares; PSSR, proton‐to‐sodium segmentation ratio; PVC, partial volume correction; STC, single target correction.

### 3.2 Results In Vivo

**FIGURE 5:**
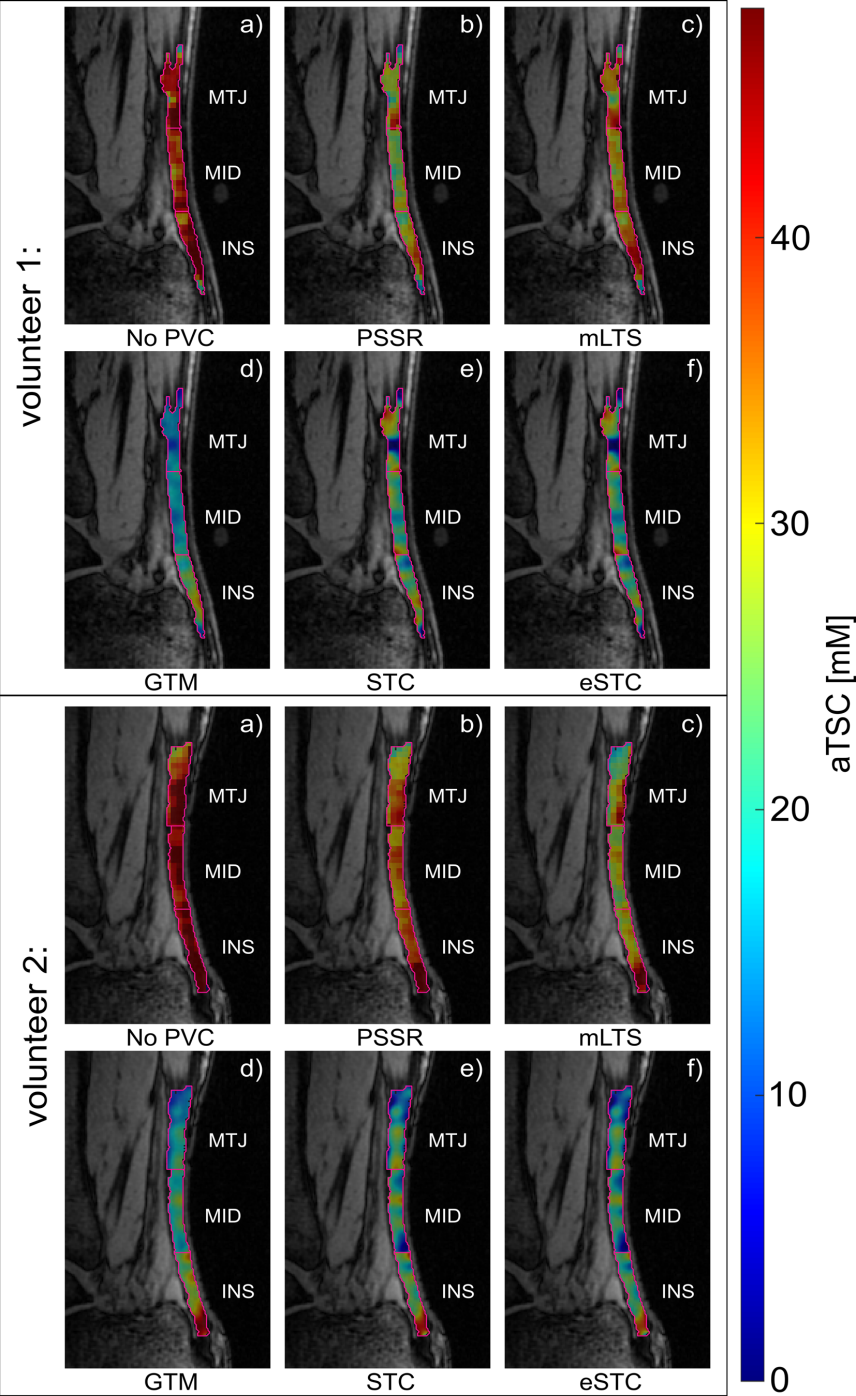
Sagittal apparent tissue sodium (aTSC) maps of the 2 mm^3^ resolution image overlayed onto the proton images of volunteer 1 and volunteer 2. Sodium and proton images were acquired with the DA‐3D‐RAD sequence. The maps were generated without PVC (a), with the PSSR (b), the mLTS (c), the GTM (d), the STC (e) and the eSTC (f). The INS, MID and MTJ subsections of the Achilles tendon are outlined and labeled accordingly.

The aTSCs of the in vivo data are presented in Table 5. The STC did not converge in the 4.5 mm^3^ images and instead reached a steady state flipping between two versions of the image after 127 iterations. The results for both are listed in Table 5. Without PVC, there was a clear increase in aTSC values with decreasing resolution. The tissue‐fraction correction methods exhibited greater differences than the spill‐over correction methods. All spill‐over correction methods showed small differences between the 1.5 and the 2 mm^3^ images. In those cases, convergence of the STC was reached after 10–11 and 13–15 iterations, respectively. The number of iterations required for STC convergence increased to 43–50 iterations for the 3 mm^3^ image. All correction methods exhibited similar behavior in both volunteers, except for the GTM in the 4.5 mm^3^ image. There, it led to an overall increase in aTSCs in Volunteer 1 and a large decrease in the MTJ in Volunteer 2. Figure 5 shows overlaid images of the corrected 2 mm^3^ sodium images on the proton image for all PVCs.

**TABLE 5:** Apparent tissue sodium contents (aTSCs) from the Achilles tendon of two volunteers were evaluated for four different nominal image resolutions.

PVC	Resolution [mm^3^]	INS [mM]	MID [mM]	MTJ [mM]	Combined [mM]	Combined absolute difference from 1.5 mm^3^ [mM]
*Volunteer 1*						
No	1.5	38.7 ± 10.4	31.3 ± 7.4	29.2 ± 8.2	35.0 ± 11.2	
	2	42.1 ± 11.7	34.0 ± 6.5	32.6 ± 8.1	38.6 ± 11.8	3.6
	3	53.3 ± 13.1	43.9 ± 5.6	46.0 ± 6.7	50.3 ± 12.5	15.3
	4.5	73.8 ± 17.3	66.9 ± 13.3	73.9 ± 10.8	73.8 ± 16.5	38.8
PSSR	1.5	32.2 ± 7.5	23.5 ± 5.3	22.1 ± 5.9	27.2 ± 8.1	
	2	36.5 ± 7.3	26.6 ± 4.1	23.8 ± 5.1	30.0 ± 7.4	2.8
	3	49.5 ± 6.4	36.1 ± 2.7	30.4 ± 3.2	38.5 ± 6.1	11.3
	4.5	63.7 ± 5.9	46.6 ± 4.6	31.5 ± 3.7	47.6 ± 5.6	20.4
mLTS	1.5	30.9 ± 7.8	24.7 ± 6.6	24.5 ± 6.8	27.8 ± 8.7	
	2	34.4 ± 9.5	26.8 ± 4.5	29.0 ± 5.9	31.1 ± 8.9	4.5
	3	40.5 ± 15.4	34.3 ± 4.4	37.0 ± 9.8	38.3 ± 12.5	12.5
	4.5	63.9 ± 81.0	49.6 ± 11.3	50.4 ± 11.8	56.9 ± 56.8	25.9
GTM	1.5	22.2 ± 5.9	14.2 ± 3.4	10.6 ± 2.9	17.0 ± 7.5	
	2	23.6 ± 5.8	14.1 ± 2.8	11.6 ± 2.8	17.9 ± 7.8	0.9
	3	26.1 ± 4.9	16.9 ± 1.1	11.9 ± 1.9	19.9 ± 7.9	2.9
	4.5	32.0 ± 4.5	21.2 ± 1.4	15.5 ± 3.0	24.8 ± 9.0	7.8
STC	1.5	22.4 ± 11.6	15.1 ± 7.9	14.6 ± 10.0	18.4 ± 11.3	
	2	23.5 ± 13.1	14.6 ± 7.5	13.9 ± 9.6	18.5 ± 12.3	0.1
	3	28.5 ± 13.2	17.9 ± 2.9	16.7 ± 5.8	22.5 ± 11.7	4.1
	4.5^a^	38.1 ± 20.4 42.4 ± 15.0	14.5 ± 7.3 24.7 ± 9.0	20.6 ± 9.4 35.9 ± 14.9	26.5 ± 19.3 42.2 ± 16.0	8.1 23.8
eSTC	1.5	21.7 ± 11.6	14.8 ± 7.9	13.9 ± 9.8	17.8 ± 11.3	
	2	21.2 ± 12.3	13.9 ± 7.5	12.6 ± 9.3	17.0 ± 11.6	0.8
	3	21.4 ± 10.7	15.0 ± 3.0	12.6 ± 5.6	17.5 ± 9.3	0.3
	4.5	27.8 ± 13.9	17.0 ± 1.9	17.6 ± 7.3	22.1 ± 11.9	4.3
*Volunteer 2*						
No	1.5	50.7 ± 13.9	37.0 ± 7.4	34.9 ± 9.4	43.9 ± 15.2	
	2	57.0 ± 13.6	41.4 ± 4.9	40.6 ± 7.5	49.9 ± 15.0	6.0
	3	65.3 ± 12.0	46.5 ± 5.2	51.1 ± 8.0	57.7 ± 8.0	13.8
	4.5	82.1 ± 22.5	64.3 ± 11.4	77.1 ± 8.0	78.1 ± 21.0	34.2
PSSR	1.5	44.8 ± 10.1	30.2 ± 5.4	27.5 ± 6.9	35.7 ± 11.1	
	2	51.4 ± 9.2	34.4 ± 3.3	27.8 ± 5.1	39.0 ± 10.1	3.3
	3	57.2 ± 7.0	34.4 ± 3.1	30.5 ± 3.0	40.3 ± 7.8	4.6
	4.5	65.7 ± 7.1	46.8 ± 3.6	27.1 ± 2.5	44.6 ± 6.7	8.9
mLTS	1.5	41.9 ± 12.0	30.3 ± 5.2	30.5 ± 8.0	36.3 ± 12.4	
	2	46.2 ± 11.6	33.5 ± 6.5	34.1 ± 5.7	40.2 ± 12.5	3.9
	3	51.4 ± 27.8	34.3 ± 6.7	37.5 ± 4.9	43.7 ± 22.5	7.0
	4.5	73.8 ± 179.8	48.6 ± 22.0	52.4 ± 16.5	62.2 ± 129.0	25.9
GTM	1.5	30.3 ± 10.1	19.3 ± 4.7	16.4 ± 5.3	24.0 ± 11.1	
	2	34.5 ± 10.2	19.4 ± 2.7	16.1 ± 3.8	25.9 ± 12.5	1.9
	3	37.6 ± 8.2	18.6 ± 1.7	16.2 ± 1.9	27.2 ± 7.9	3.2
	4.5	34.8 ± 6.9	15.9 ± 0.7	7.3 ± 0.9	22.8 ± 14.1	1.2
STC	1.5	26.6 ± 13.9	18.2 ± 8.2	17.3 ± 10.4	22.2 ± 13.2	
	2	32.0 ± 15.0	18.3 ± 5.9	16.2 ± 8.5	24.5 ± 14.8	2.3
	3	38.6 ± 13.7	21.8 ± 5.2	21.4 ± 4.8	30.0 ± 14.5	7.8
	4.5^a^	49.4 ± 17.6 50.7 ± 16.7	33.0 ± 6.4 36.3 ± 6.9	33.1 ± 8.6 34.9 ± 9.2	41.3 ± 17.1 43.3 ± 16.5	21.1 19.1
eSTC	1.5	25.6 ± 14.0	17.8 ± 8.3	16.5 ± 10.4	21.4 ± 13.2	
	2	28.4 ± 13.6	17.0 ± 5.8	14.8 ± 8.4	22.1 ± 13.3	0.7
	3	28.6 ± 10.5	17.8 ± 4.7	16.8 ± 4.6	22.9 ± 10.7	1.5
	4.5	33.6 ± 12.4	26.4 ± 6.5	24.2 ± 9.2	29.7 ± 12.1	7.7

*Note*: The results were obtained once without PVC and with five different PVC methods. The listed errors are the standard deviations across the ROI of each image. The absolute difference between the aTSC results of the different resolutions to the highest 1.5 mm^3^ resolution image is shown in mM.

Abbreviations: PSSR: proton‐to‐sodium segmentation ratio, mLTS: modified least trimmed squares, GTM: geometric transfer matrix, STC: single target correction, eSTC: estimated single target correction, INS: insertion point into the calcaneus, MID: middle portion of the tendon, MTJ: myotendinous junction, aTSC: apparent tissue sodium content.

^a^ There was no conversion reached with the STC in the 4.5 mm^3^ image. Instead, a steady state was reached after 127 iterations, that switched between two versions of the image. The results with both versions are shown.

## 4 Discussion

Five different PVC methods were evaluated for their application to the Achilles tendon using a simulated dataset and were applied in vivo measurements from two volunteers. A substantial impact of PVEs on aTSC determination was demonstrated, which was improved by all methods. The PSSR method was modified to incorporate signals from surrounding tissues. The STC method was adapted for sodium MRI by combining it with the PSF simulation from Niesporek et al. [23] An alternative method for voxel‐wise spill‐over correction was proposed with the eSTC. The simulation illustrated the quantitative and visual effects of the PVCs. In vivo measurements of two volunteers at four resolutions were used to obtain aTSC values and replicated the simulation's results.

The simulation results showed that aTSC calculations improved with PVC overall. Deviations tended to present as overestimated concentrations. This overestimation resulted from PVEs of the reference phantoms, which have been observed previously [23]. Since the reference phantoms were not surrounded by any signal, all uncorrected phantom values were underestimated when affected by PVEs. Because they were used for aTSC calculation, this underestimation resulted in an overestimation of the tissues. This effect was also apparent in the in vivo images, where aTSC values were calculated with each PVC. Without PVC, large differences in aTSC values were observed at different resolutions, with an overall increase in aTSCs at lower resolutions. In vivo aTSC values without PVC were lower than previously published values, with a mean value of 82.2 ± 13.9 mM [6] with a 2 mm^3^ isotropic resolution. These differences may be due to different applications of PVC methods. Kamp et al. [6] applied the PSSR method without the extension that includes the surrounding signal. Since they assumed zero signal in the surroundings, the PSSR method corrected to higher values as opposed to lower values with the extension. Additionally, they did not correct the signal of the reference phantoms, which could have led to overestimation as discussed above. In the future, larger phantoms may be preferable, as they are less susceptible to PVEs. However, there was limited space in the FOV of the coil used here, which limited the possible phantom size.

Of all the correction methods, the PSSR and mLTS produced the largest differences from the ground truth and RMSEs in the simulation. Correction of tissue‐fraction artifacts was thereby less effective, suggesting that the spill‐over effect dominated the images in this study. Both the simulation and the in vivo images reflected radial acquisition with corresponding PSFs [23], which have inherently larger FWHMs [22] compared to Cartesian acquisitions. In those cases, the PSF is often considered negligible [25] and the mLTS and PSSR methods would likely perform better. Consistent with the simulation, the in vivo measurements showed larger differences between the various resolutions with the tissue‐fraction corrections. Additionally, the mLTS resulted in a larger SD than the aTSC value itself in the 4.5 mm^3^ image. This suggests that the mLTS method may produce outliers.

There were three methods for correcting spill‐over artifacts: the GTM, STC and eSTC. All three led to low differences in the simulation (Tables 3, 4 and Figure 3). Among the spill‐over corrections, the GTM produced the largest differences to the ground truth in the simulation (Figure 3). However, these decreased at a lower SNR and the GTM had the lowest RMSEs in the Monte Carlo simulation, indicating that there were larger outliers with STC and eSTC than with the GTM. This suggests that the GTM is less sensitive to noise and more robust at low SNRs.

The higher deviations compared with the GTM and the other spill‐over methods are likely due to the simulated coil sensitivity, resulting in weaker signals at greater distances from the coil. This gradient was present within a tissue and influenced the mean signals used to calculate the corrected values with the GTM. This effect is visible in (Figure 4e), particularly in the INS. Note that when excluding the inhomogeneous coil sensitivity from our simulation (Figure S1 and Table S1), the mean aTSCs with the GTM were much more similar to the ground truth. In vivo, the GTM shows deviations from the 1.5 mm^3^ image, primarily in the INS, except for the 4.5 mm^3^ image of Volunteer 2, where it produces a very similar combined value, but a strong deviation in the MTJ. This could also be caused by coil sensitivity, since the relative position of the foot and coil may have varied slightly between volunteers, and the GTM appears sensitive to these variations. One potential solution for this could be the use of volume coils, which have a more homogeneous coil sensitivity [24]. Their use resulted in a higher accuracy of the GTM in previous studies [23]. For example, Lott et al. [26] simulated spill‐in from blood signal into muscle tissue and Gast et al. [46] simulated the whole lower leg. Both demonstrated a good recovery of muscle signals [26, 46]. Niesporek et al. [23] demonstrated accurate aTSCs recovery in phantom simulations and measurements, as well as in the human brain, using the GTM [23].

The STC demonstrated good recovery of the mean signals and the low SDs without noise indicated that signal variations were preserved in the simulation. Nevertheless, the in vivo results with lower resolutions differed from the simulation results. While the difference between the 1.5 and 2.0 mm^3^ images was low, the deviation in the 3.0 mm^3^ image was greater and the number of iterations necessary for convergence increased drastically. No clear results were possible for the 4.5 mm^3^ images since there was no convergence. Sari et al. [36] evaluated a simulated PET dataset with the STC method at approximately 2 mm^3^ resolution. Their in vivo acquisition was achieved using a 2.5 mm pixel size and 3 mm slice thickness. Convergence was reached in both cases after 10 iterations [36]. These voxel sizes were similar to the 2.0 and 3.0 mm^3^ images in this study, where the method converged. Therefore, the method may have problems with larger voxel sizes.

The eSTC produced the most accurate concentration recovery in both the simulation and in vivo. There were low differences up to the 3.0 mm^3^ image and it resulted in comparatively low deviations with the 4.5 mm^3^ images at 4.3 and 7.7 mM. Even though the GTM had a closer overall concentration in the 4.5 mm^3^ image of volunteer 2, there was a stronger variation in the individual parts, especially the MTJ. Although the eSTC led to relatively large SDs in vivo, these do not necessarily imply lower accuracy, as physiological variations would also lead to higher SDs. These variations were preserved with the eSTC, as can be seen in Figure 5. The inhomogeneous sodium distribution in the tendon was also visible in 7 T sodium images [13, 14]. The simulated lower SDs without noise also indicate a good preservation of variations, although it was more sensitive to noise (Figure 3b) and outliers, especially at a lower SNR. One downside of this method is that it is limited to tissues large enough to have remaining voxels after erosion. For smaller tissues, the uneroded values can be used for estimation, but this would lead to lower accuracy. Moreover, small structures at tissue interfaces may be disregarded if they are smaller than the eroded zone. This could result in overlooking spill‐over contributions. Yet, this did not occur in the case of the AT. The current implementation focused on correcting the AT and neglected the accuracy of the surrounding structures. For applications where the surrounding structures are relevant, the method would have to be applied separately to all relevant structures. Another option would be to combine the eSTC method with the multi‐target correction (MTC) proposed by Erlandsson et al. for PET [29]. This method includes a first iteration with the GTM, followed by an STC application [47].

This study focused on the application to the AT, but the results can be applied to other structures. However, factors such as coil sensitivity, tissue size, the importance of regional differences, and the number of compartments, should be considered. Surface coils are commonly used for structures such as cartilage [2, 28, 42], skin [40] or muscles [46, 48, 49], and could benefit from the application of the eSTC. For abdominal [26, 50] or brain [19, 51] measurements, the GTM may be sufficient, but the eSTC would better preserve regional differences.

The limitations of this study must be acknowledged. First, the simulation was designed to reflect a previous study's resolution and SNR [6]. Since the simulation's results could be validated against the ground truth, other resolutions were not included.

In vivo, B1 inhomogeneities were not considered, which can affect aTSC determination [52, 53]. Although several methods exist for B1 correction, such as the double‐angle [1, 53], and the Bloch‐Siegert shift method [54], they either significantly increase measurement time or result in a reduced SNR [53]. Additionally, the true coil sensitivity can only be approximated by NaCl solution, since the dielectric properties and coil loading [55] of the solution differ from those of human tissue. However, acquiring individual sensitivity maps would further increase the measurement time [1]. Furthermore, T_1_‐relaxation weighting was present in this study due to the short TR, which required correction [2, 45]. Since the AT is generally a low‐SNR tissue [14], large numbers of projections are necessary at clinical field strengths [6]. Thus, the TR was reduced to enable in vivo measurements at a reasonable acquisition time. Additionally, relaxation times were not determined due to the long acquisition time. Therefore, literature values were used [6] for relaxation correction [45]. Inaccurate relaxation times can also affect the FWHM of the PSF [23]. However, Niesporek et al. [23] have simulated the impact on the correction accuracy with the GTM, which is relatively small [23]. Lastly, the PSF of the surroundings for the STC and eSTC methods was modeled using the mean relaxation times of all surrounding tissues, resulting in an averaged PSF. Nevertheless, the simulation results indicated that this was a sufficient estimate. This is not an issue for PET applications, because the same Gaussian PSF can be used for all tissues [36]. Using individual PSFs may also increase the number of iterations or contribute to the convergence issues.

Overall, this study demonstrated that all PVC methods could increase the accuracy of aTSC determination. The eSTC method proved to be the most dependable for AT measurements with a radial sequence and a surface coil.

## 5 Conclusion

Five different PVC methods were compared with in silico data and in vivo measurements for their application to sodium MRI of the Achilles tendon. All methods improved the accuracy of determining apparent tissue sodium content, with spill‐over corrections proving more effective than tissue‐fraction corrections. The eSTC method introduced here led to the most accurate results.

## Funding

This work was supported by Jürgen Manchot Stiftung; Deutsche Forschungsgemeinschaft, 530863408.

## Supporting information


**Figure S1:** Monte Carlo simulation results with homogeneous coil sensitivity. Mean differences per voxel in the Achilles Tendon between the partial volume effect corrected image and the ground truth at an SNR in the MID (myotendinous junction) of 10 (a) and 5 (b). The ground truth was voxel‐wise subtracted from the image before averaging over the respective region of interest. The standard deviations over the 100 noise iterations are indicated.
**Table S1:** Simulated results with homogeneous coil sensitivity. Mean differences per voxel (Δ) between the partial volume effect corrected image without noise and the ground truth. Positive values represent overestimated concentrations, negative values underestimated concentrations. The standard deviation over the respective region of interest is given.

## Data Availability

The data that support the findings of this study are available from the corresponding author upon reasonable request.
