# Supplementary material for: Evaluation of Partial Volume Correction Techniques for Sodium MRI of the Achilles Tendon
Source: Magn Reson Med. 2025 Nov 27;95(4):2180–93. doi: 10.1002/mrm.70208 (PMC12850615; doi:10.1002/mrm.70208)
Supplement: Supplementary file 1 — Figure S1: Monte Carlo simulation results with homogeneous coil sensitivity. Mean differences per voxel in the Achilles Tendon between the partial volume effect corrected image and the ground truth at an SNR in the MID (myotendinous junction) of 10 (a) and 5 (b). The ground truth was voxel‐wise subtracted from the image before averaging over the respective region of interest. The standard deviations over the 100 noise iterations are indicated. Table S1: Simulated results with homogeneous coil sensitivity. Mean differences per voxel (Δ) between the partial volume effect corrected image without noise and the ground truth. Positive values represent overestimated concentrations, negative values underestimated concentrations. The standard deviation over the respective region of interest is given. [file MRM-95-2180-s001.docx]

Supporting Information


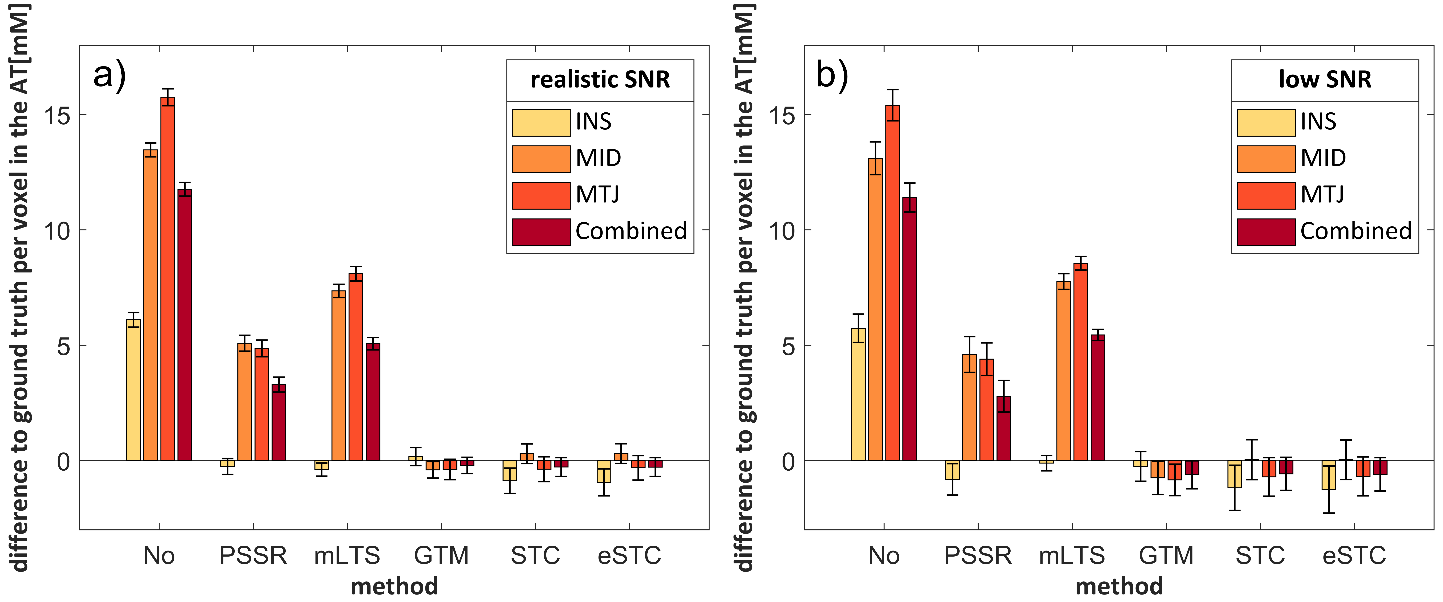


Supporting Information Figure S1: Monte Carlo simulation results with homogeneous coil sensitivity. Mean differences per voxel in the Achilles Tendon between the partial volume effect corrected image and the ground truth at an SNR in the MID (myotendinous junction) of 10 a) and 5 b). The ground truth was voxel-wise subtracted from the image before averaging over the respective region of interest. The standard deviations over the 100 noise iterations are indicated.

Supporting Information Table S1: Simulated results with homogeneous coil sensitivity. Mean differences per voxel (Δ) between the partial volume effect corrected image without noise and the ground truth. Positive values represent overestimated concentrations, negative values underestimated concentrations. The standard deviation over the respective region of interest is given.

| **PVC** | **Δ INS [mM]** | **Δ MID [mM]** | **Δ MTJ [mM]** | **Δ Combined [mM]** |
| --- | --- | --- | --- | --- |
| **No** | 6.34 ± 7.18 | 13.68 ± 2.48 | 15.91 ± 2.87 | 11.96 ± 6.12 |
| **PSSR** | 0.07 ± 8.38 | 5.38 ± 2.42 | 5.11 ± 1.79 | 3.58 ± 5.64 |
| **mLTS** | -0.42 ± 5.49 | 7.17 ± 1.68 | 7.82 ± 2.21 | 4.91 ± 5.09 |
| **GTM** | 0.54 ± 6.13 | -0.06 ± 1.36 | -0.07 ± 1.69 | 0.13 ± 3.72 |
| **STC** | -0.52 ± 2.45 | 0.63 ± 0.99 | -0.12 ± 1.89 | 0.03 ± 1.93 |
| **eSTC** | -0.59 ± 2.25 | 0.64 ± 0.98 | -0.03 ± 1.88 | 0.04 ± 1.82 |
